# Aids to Obstetrics, Aids to Gynæcology

**Published:** 1886-02

**Authors:** 


**Aids to Obstetrics** (double part). By SAMUEL NALL, B. A., M. B. **Aids to Gynæcology.** By Alfred S. Gubb, L. R. C. P., M. R. C. S. New York and London: G. P. Putnam's Sons. 1885.

The above belong to the Students' Aid Series, now being issued by the Putnams. They both contain the leading facts of their respective subjects, in a form convenient to be a help to the student in preparing for examination. They are not intended to interfere in any way with the systematic reading on the subject. We believe that their study will be an aid to the student in impressing upon his memory the salient features of the subjects.

